# A model of mitochondrial superoxide production during ischaemia-reperfusion injury for therapeutic development and mechanistic understanding

**DOI:** 10.1016/j.redox.2024.103161

**Published:** 2024-04-24

**Authors:** Annabel Sorby-Adams, Tracy A. Prime, Jan Lj Miljkovic, Hiran A. Prag, Thomas Krieg, Michael P. Murphy

**Affiliations:** a MRC Mitochondrial Biology Unit, University of Cambridge, The Keith Peters Building, Cambridge, CB2 0XY, UK; b Department of Medicine, University of Cambridge, Hills Road, Cambridge, CB2 0QQ, UK

**Keywords:** Ischaemia-reperfusion injury, Mitochondria, Succinate, Malonate, Complex I, Reverse electron transport

## Abstract

Ischaemia-reperfusion (IR) injury is the paradoxical consequence of the rapid restoration of blood flow to an ischaemic organ. Although reperfusion is essential for tissue survival in conditions such as myocardial infarction and stroke, the excessive production of mitochondrial reactive oxygen species (ROS) upon reperfusion initiates the oxidative damage that underlies IR injury, by causing cell death and inflammation. This ROS production is caused by an accumulation of the mitochondrial metabolite succinate during ischaemia, followed by its rapid oxidation upon reperfusion by succinate dehydrogenase (SDH), driving superoxide production at complex I by reverse electron transport. Inhibitors of SDH, such as malonate, show therapeutic potential by decreasing succinate oxidation and superoxide production upon reperfusion. To better understand the mechanism of mitochondrial ROS production upon reperfusion and to assess potential therapies, we set up an *in vitro* model of IR injury. For this, isolated mitochondria were incubated anoxically with succinate to mimic ischaemia and then rapidly reoxygenated to replicate reperfusion, driving a burst of ROS formation. Using this system, we assess the factors that contribute to the magnitude of mitochondrial ROS production in heart, brain, and kidney mitochondria, as well as screening for inhibitors of succinate oxidation with therapeutic potential.

## 1 Introduction

When blood flow to an organ is disrupted, such as during myocardial infarction, stroke, organ transplantation or elective surgery, rapid reperfusion of the ischemic tissue is essential to minimise cell death [[1], [2], [3]]. However, even though reperfusion is essential to salvage cells at risk of conversion to permanent infarction, the restoration of blood flow paradoxically causes further tissue injury [4,5]. This ischaemia-reperfusion (IR) injury greatly exacerbates tissue damage and therapeutic approaches to minimise IR injury are urgently required [6,7].

IR injury is initiated by the mitochondrial production of reactive oxygen species (ROS) [8]. During ischaemia, oxidative phosphorylation stops, resulting in loss of ATP, ion dyshomeostasis, calcium overload, acidosis, and accumulation of the mitochondrial metabolite succinate [8,9]. Immediately upon reperfusion, the reintroduction of oxygen (O_2_) drives the rapid reoxidation of succinate by succinate dehydrogenase (SDH) [10]. This results in a reduced Coenzyme Q (CoQ) pool and an elevated protonmotive force (Δp) across the mitochondrial inner membrane that drives reverse electron transport (RET) at complex I, generating a burst of superoxide (O_2_^•−^) upon reperfusion [8]. The O_2_^•−^ is rapidly dismutated to hydrogen peroxide (H_2_O_2_) by manganese superoxide dismutase [11,12], causing the oxidative damage that, in concert with hyperosmolarity, acidosis, and intracellular calcium accumulation, initiates IR injury.

This ROS burst can be blocked by inhibiting complex I or SDH, by uncoupling mitochondria, or by preventing the high membrane potential formation via inhibition of complex IV by H_2_S [8,[13], [14], [15], [16]]. Approaches with therapeutic potential in animal models of heart attack and stroke include the use of the competitive SDH inhibitor malonate, either unmodified, or as an esterified pro-drug [8,9,13,14], and site-specific suppressors of superoxide production (S1QELs) that block RET ROS production at complex I without affecting respiration [17,18]. While these approaches show therapeutic promise, a simpler, isolated mitochondria model is required because many of the details of mitochondrial ROS production upon reperfusion are challenging to assess *in vivo*.

An *in vitro* model would also facilitate the screening of other potential therapeutic approaches. The role of mitochondria in IR injury has been extensively explored using isolated mitochondrial systems (see e.g. [[19], [20], [21], [22], [23]]. However, these studies were carried out before the emergence of succinate-driven ROS production by RET at complex I as an important contributor to IR injury. Therefore, in this study we established a model of how succinate may contribute to IR injury at reperfusion in which we incubate isolated mitochondria under conditions that mimic ischaemia and then rapidly reoxygenate the system to replicate reperfusion. In this system, we can simultaneously measure mitochondrial ROS production and O_2_ consumption, enabling us to both elucidate mechanistic aspects of mitochondrial ROS synthesis and screen putative therapies. To illustrate this, we explored the impact of ADP concentration and pH on the generation of ROS upon reoxygenation and also screened a number of potential compounds to assess their ability to decrease the mitochondrial ROS burst upon reoxygenation.

## 2 Methods

### 2.1 Animals

Wild-type female Wistar rats (Charles River) aged 9–12 weeks were used for all experiments. Animals were housed in individually ventilated cages with *ad lib* access to water and commercial rat chow. Animals were humanely killed by cervical dislocation, following which tissues of interest were rapidly harvested. All animal work was carried out according to the United Kingdom Animals (Scientific Procedures) Act 1986 and University of Cambridge Animal Welfare Policy and was approved by the University of Cambridge Animal Welfare and Ethical Review Body.

### 2.2 Mitochondrial isolation

Rat heart mitochondria were isolated by differential centrifugation based on previously described methods [24]. Immediately following excision, tissue was finely cut and washed in STEB buffer (250 mM sucrose, 10 mM Tris-HCl, 1 mM EGTA, 0.1% w/v fatty acid-free bovine serum antigen (BSA), pH 7.4). Tissue pieces were homogenised in an all glass Dounce homogeniser, centrifuged twice (700×*g*, 5 min, 4 °C) and filtered through pre-wet muslin. Crude mitochondria were pelleted by centrifugation (10,000×*g*, 5 min, 4 °C), supernatant removed and washed once with STEB then centrifuged again (10,000×*g*, 5 min, 4 °C). Pelleted mitochondria were resuspended in STE buffer (without BSA) and the protein concentration determined using a bicinchoninic acid (BCA) assay kit with BSA as the standard. Heart mitochondria were stored on ice and used within 4 h.

Rat brain mitochondria were isolated by Percoll gradient centrifugation [25]. Brains were excised, infratentorial structures removed, weighed and isolation buffer (IB, 320 mM sucrose, 1 mM EDTA, 10 mM Tris-HCl, pH 7.4) added (10% w/v). Brains were homogenised in an all glass Dounce homogeniser and centrifuged (1300×*g*, 4 min, 4 °C). Supernatant was retained and the pellet resuspended and centrifuged at 21,000×*g* (10 min, 4 °C). The resultant pellet was resuspended in 8 mL 15% Percoll, overlaid on a Percoll step gradient (7 mL 23% and 7 mL 40%), centrifuged (30,700×*g*, 5 min, 4 °C) and the band between the 23 and 40 % Percoll steps resuspended in 4 × volumes of IB and centrifuged (16,700×*g*, 10 min, 4 °C), generating a loose pellet. Supernatant was aspirated, 0.5 mL per brain of 10 mg/mL BSA in IB was added then centrifuged (6900×*g*, 10 min, 4 °C) to produce a firm pellet. The pellet was resuspended in IB and protein concentration determined using a BCA assay. Brain mitochondria were stored on ice and used within 5 h.

Rat kidney mitochondria were isolated as described for the heart, with kidneys sliced lengthways to enable the calyx to be removed prior to homogenisation.

### 2.3 Measurement of mitochondrial O_2_ consumption and ROS production

For simultaneous measurement of O_2_ consumption and ROS production we used a two-chamber, high resolution O2k Oxygraph equipped with a 525 nm fluorescence LED module (Oroboros Instruments, Innsbruck, Austria). ROS production was determined from the fluorescence of resorufin, generated from the H_2_O_2_ -dependent oxidation of Amplex Red (N-acetyl-3,7-dihydroxyphenoxazine) by horseradish peroxidase (HRP) [[26], [27], [28], [29]]. The response to H_2_O_2_ was calibrated in the presence of mitochondria, fatty-acid free BSA, HRP, superoxide dismutase (SOD) and Amplex Red at the beginning and end of each set of experiments by up to 20 titrations of 200 pmol H_2_O_2_ (2 μM final concentration). Calibration at air saturation was carried out prior to experimentation and all data was corrected for background instrumental O_2_ flux in accordance with the manufacturer's instructions and zero O_2_ determined when all O_2_ had been depleted by mitochondrial respiration in the closed chamber. For each experiment, duplicate samples were run in both chambers as technical replicates. A minimum of 3 independent mitochondrial preparations were assessed to generate biological replicates, unless stated otherwise.

Mitochondria (0.5 mg protein/mL) were incubated in each chamber of the O2k Oxygraph containing 2 mL KCl buffer (120 mM KCl, 10 mM HEPES, 1 mM EGTA, pH 7.2) supplemented with 40 μg/mL SOD (Cu, Zn, Sigma S8160), 20 μg/mL HRP (Sigma P8250), 200 μg/mL fatty acid free BSA (Sigma A3803), and 12.5 μM AmplexRed (Invitrogen A12222) while stirring (750 rotations/min) at 37 °C. Upon addition of the mitochondria, chambers were closed and after 2 min succinate (10 mM, Sigma S3674) was added to each chamber. Respiration was continued until anoxia, and after either 5 or 10 min of anoxia, the chambers were opened to the atmosphere with stirring to reoxygenate the incubation under dark room conditions for an additional 10 min. Some experiments were carried out at pH 6.5, in which case the H_2_O_2_ calibration was done at that pH. For some experiments either rotenone (4 μg/mL, Santa Cruz SC203242) or FCCP (500 nM, Sigma C2920) were added at the beginning of the incubation. In other experiments, ADP (Sigma A2754) was added to the chamber after 5 min of anoxia and for these experiments the KCl buffer was supplemented with 5 mM phosphate and the pH readjusted. Other compounds of interest were also added after 5 min anoxia: disodium malonate (DSM, 10 mM, Aldrich 63,419), butylmalonate (20 mM, TCI B4414), MitoQ (2.5 μM, a gift from MitoQ Inc), Cyclosporin A (1.6 μM, Sigma 30,024), Metformin HCl (10 mM, LKT Laboratories Inc, M2076), and S1QEL 1.1 (2.5 μM, Sigma SML1948)).

### 2.4 Measurement of dose response curves for inhibition of RET ROS

A dose response curve to determine concentrations of compounds that affect RET in isolated heart mitochondria using a 96 well screen assay was performed as described previously [29]. For this, rat heart mitochondria (0.7 mg protein/mL) were added to a final volume of 200 μL of the same incubation medium described in Section 2.3, supplemented with 10 mM succinate, along with the compounds of interest diluted in water (DSM, butylmalonate, metformin) or DMSO (MitoQ, Cyclosporin A, S1QEL 1.1). The following concentrations were tested for each of the inhibitors: DSM (0–10 mM), Metformin (0–10 mM), butylmalonate (0–20 mM), MitoQ (0–5 μM), S1QEL 1.1 (0–5 μM) and Cyclosporin A (0–1.6 μM). The production of H_2_O_2_ was then measured using a fluorescence plate reader (SpectraMax Gemini XS, Molecular Devices) with excitation 570 nm, emission 585 nm, at 37 °C with shaking at the start and before each reading for 10 min. The mean slopes of the increase in fluorescence ± SD were determined in quadruplicate at each concentration.

### 2.5 Statistical analyses

Statistical analysis was carried out using GraphPad Prism (v10, GraphPad Software, Massachusetts, USA). For each experiment, the raw resorufin signal was converted to [H_2_O_2_] (*μ*M) by interpolating the amplitude of the fluorescent signal with the corresponding H_2_O_2_ calibration from the given experimental day. The [O_2_] (*μ*M) and interpolated [H_2_O_2_] (*μ*M) were extrapolated from each experiment at 1-min intervals using custom MATLAB (vR2023a, Massachusetts, USA) code. Technical duplicates were averaged and exported for final analysis. Parametric one-way analyses of variance (ANOVA) with Tukey's post-hoc tests or unpaired t-tests were performed to compare [H_2_O_2_] and [O_2_] for each experimental condition. The number of technical and biological replicates are defined in each figure legend and expressed as mean ± SEM. A p value ≤ 0.05 was considered statistically significant throughout.

## 3 Results and discussion

### 3.1 Establishing an isolated mitochondrial model of IR injury

**Fig. 1:**
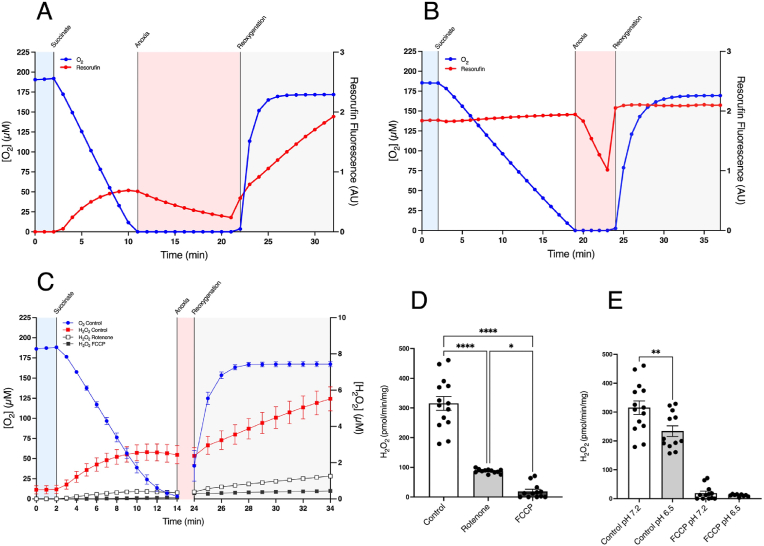
Modelling IR ROS production in isolated heart mitochondria. (A) An exemplar experiment showing heart mitochondria with simultaneous measurement of [O_2_] (blue) and resorufin fluorescence (red), generated by the H_2_O_2_-dependent oxidation of Amplex Red. Where indicated, succinate (10 mM) was added to the sealed chamber which led to anoxia. After 10 min anoxia the stirred chamber was opened to the atmosphere which led to immediate reoxygenation. (B) Mitochondria were incubated as in (A) with resorufin (6 μM) and [O_2_] measured simultaneously. (C) Mitochondria were incubated as described in (A) in the presence of FCCP (500 nM) or rotenone (4 μg/mL). (D) The initial rates of H_2_O_2_ production determined over the first 10 min post reoxygenation. (E) The rate of H_2_O_2_ production after addition of succinate to the closed chamber as a function of [O_2_]. * = p < 0.05, **** = p < 0.0001. Data are means ± SEM of *n* = 12–14 experiments each on a separate mitochondrial preparation.

To establish an *in vitro* model of mitochondrial ROS production during IR we incubated heart mitochondria in an O2k-FluoRespirometer and measured [O_2_] (blue) and resorufin fluorescence (red) simultaneously, as illustrated in Fig. 1A. Mitochondria respiring after addition of succinate in this closed system decreased the [O_2_] to anoxic levels due to the low apparent *K*_m_ of cytochrome oxidase [30], mimicking the situation during ischaemia [31] (Fig. 1A). The mitochondria could be maintained under anoxia until the rapid onset of reperfusion upon restoration of blood flow was mimicked by opening the stirred chamber (reoxygenation) to atmospheric oxygen (Fig. 1A).

Measurement of resorufin fluorescence showed an increase of Amplex Red oxidation upon addition of succinate due to production of H_2_O_2_ from RET at complex I producing mitochondrial O_2_^•−^ within the mitochondrial matrix that was then dismutated to H_2_O_2_ followed by its diffusion from mitochondria (Fig. 1A). Upon anoxia, H_2_O_2_ generation ceased while the fluorescence of the accumulated resorufin decreased, due to its reduction to non-fluorescent dihydroresorufin by anaerobic mitochondria [26] (Fig. 1B). The fluorescence yield of resorufin is unaffected by [O_2_] [29]. Upon reoxygenation, the accumulated dihydroresorufin was immediately reoxidised back to resorufin, restoring the fluorescence intensity to that observed prior to anoxia (Fig. 1A and B). The efflux of H_2_O_2_ from mitochondria re-established itself upon reoxygenation to the maximum rate immediately after addition of succinate prior to anoxia and sustained this rate (Fig. 1A). In subsequent experiments we omitted the anoxic resorufin/dihydroresorufin reduction/oxidation cycle for clarity, calibrated the H_2_O_2_ response with authentic H_2_O_2_ and averaged multiple replicates (Fig. 1C and D). The generation of H_2_O_2_ upon reoxygenation was blocked by the complex I inhibitor rotenone and by the uncoupler FCCP (Fig. 1C and D), consistent with succinate driving H_2_O_2_ production by RET at complex I upon reperfusion [29]. The initial H_2_O_2_ production decreased with time (Fig. 1A) due to the [O_2_] decreasing in the closed chamber following the addition of succinate (Fig. 1E) [12,29]. In contrast, the rate of H_2_O_2_ generation after reoxygenation in the open chamber increased over the first minute as the [O_2_] increased and then remained stable because the [O_2_] remained high (Fig. 1A). This mirrors the increase in [O_2_], although the slower response of the Clark-type electrode to step changes in [O_2_] compared to the fluorescent read out introduces a slight lag. We also note that here we reoxygenate the mitochondria with air, thus the [O_2_] will reach a maximal level within a minute or so of mixing. This contrasts with the reperfusion of ischaemic tissues *in vivo* where the [O_2_] following reperfusion will be lower and its rate of increase will depend on many factors such as blood flow and extent of reperfusion. In all cases, as mitochondrial H_2_O_2_ production by RET at complex I upon reperfusion depends linearly on [O_2_] [29], the H_2_O_2_ production upon reperfusion will be proportional to the local [O_2_]. As ROS production by RET is critically dependent on a high Δp [29] it is important to demonstrate that these incubations do not lead to mitochondrial uncoupling. To assess this, we measured coupled respiration rate prior to anoxia and reoxygenation and compared this rate to that afterwards by closing the incubation chamber to enable respiration rate to be measured (Supplemental Fig. 1A). There was no increase in this sensitive marker of mitochondrial uncoupling [32], indicating minimal uncoupling occurred. In addition, the fold-stimulation of respiration by uncoupler was similar after anoxia and reoxygenation and compared to that prior to anoxia (Supplemental Fig. 1B). Consistent with these findings, the mitochondrial ROS generation upon reoxygenation is blocked by both rotenone and FCCP (Fig. 1), indicating that this ROS production is via RET at complex I which further shows that the mitochondria are well coupled as RET requires a near maximal Δp [29]. A further important consideration is whether the duration of anoxia will impact on the extent of ROS production upon reoxygenation. *In vivo*, a considerable duration of warm ischaemia is required to prime the tissue for ROS production upon reperfusion, through the accumulation of succinate and the depletion of the adenine nucleotide pools [33,34]. In contrast, in our *in vitro* model we add succinate and omit adenine nucleotides to mimic the situation occurring at the end of ischaemia, just prior to reperfusion. Even so, it is important to demonstrate that the exposure of mitochondria to prolonged anoxia does not impact the mechanism of ROS production. To assess this, we measured the rate of RET H_2_O_2_ production by complex I prior to anoxia and then 5 and 10 min after anoxia (Supplemental Fig. 1C). As expected, doubling the duration of anoxia *in vitro* does not alter the rate of H_2_O_2_ production, although there is a small decrease in this rate post anoxia/reoxygenation compared to that before. In summary, we have generated an *in vitro* system that mimics the *in vivo* mitochondrial ROS burst that occurs in tissues upon reperfusion.

### 3.2 Modelling the effect of ischaemic changes on RET ROS

**Fig. 2:**
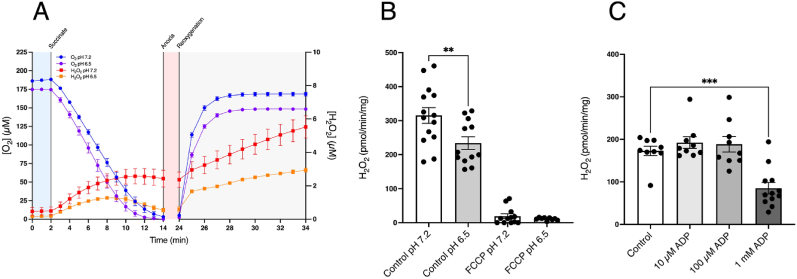
Effect of low pH and ADP on mitochondrial RET ROS. (A) Heart mitochondria were incubated as in Fig. 1A at pH 7.2 or 6.5. (B) The initial rates of H_2_O_2_ production determined over the first 10 min post reoxygenation at pH 7.2 or 6.5 ± FCCP (500 nM). (C) Heart mitochondria were incubated as in Fig. 1A with 5 mM phosphate and ADP (10 μM, 100 μM, or 1 mM) added after 5 min anoxia and the initial rates of H_2_O_2_ production determined over the first 10 min post reoxygenation. ** = p < 0.01, *** = p < 0.001. Data are means ± SEM of *n* = 9–14 experiments each on a separate mitochondrial preparation.

We have shown that in our isolated mitochondria system succinate oxidation upon reoxygenation leads to a burst of H_2_O_2_ production driven by RET. However, the mitochondrial environment upon reperfusion of ischemic tissue may be different from that in Fig. 1. Among these changes is a decrease in local pH during ischaemia to approximately 6.5 [35,36]. To see how pH affected RET, we incubated heart mitochondria at pH 6.5. Succinate oxidation and subsequent reoxygenation led to a burst of H_2_O_2_ qualitatively similar to that at pH 7.2 (Fig. 2A) but the rate was about 40% lower (Fig. 2B). Thus, a local drop in pH will somewhat decrease ROS production upon reperfusion.

RET driven ROS production requires a near maximal Δp, as described above, thus uncoupling with FCCP abolishes RET ROS production as seen in Fig. 1D. The presence of ADP can also decrease RET by utilising Δp to generate ATP [37]. Thus, the presence of ADP during the reperfusion of ischemic tissues would potentially abolish pathological ROS production by RET. To assess the [ADP] necessary to do this, we added 10 μM, 100 μM and 1 mM ADP after 5 min of anoxia and then after an additional 5 min reoxygenated the chamber and measured H_2_O_2_ production (Fig. 2C). The presence of phosphate decreased RET H_2_O_2_ production compared to its absence (Fig. 1D). The dependence of RET on [ADP] demonstrates that an [ADP] of 1 mM is required to block RET. During ischaemia, adenine nucleotides degrade by the action of the purine nucleotide cycle [8,13,38]. In particular, in mouse, pig and human heart tissue the sum of ATP and ADP is largely abolished during ischaemia with <0.5 nmol/g wet weight remaining in the mouse heart after 30 min warm ischaemia [38]. As the amount of intracellular water that the mouse heart contains is ∼615 μL/g wet weight [39], this corresponds to an [ADP] of ∼800 nM. Thus, the [ADP] in tissue after ischaemia *in vivo* is far below the levels that would affect RET ROS production upon reperfusion.

### 3.3 RET ROS during IR in brain, kidney and liver mitochondria

**Fig. 3:**
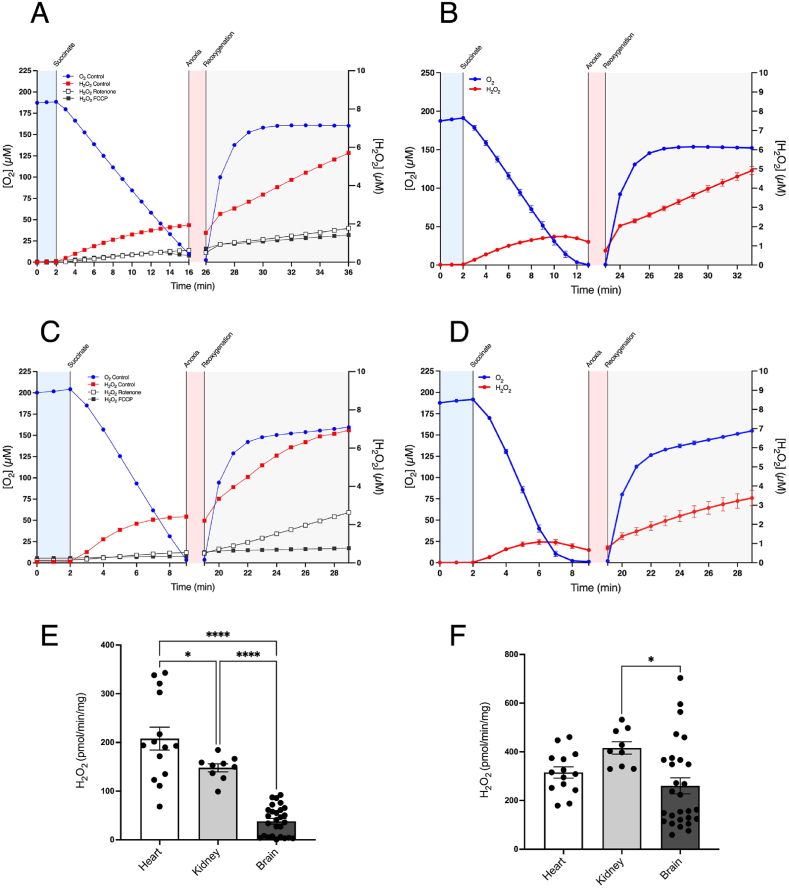
Respiration and ROS production in isolated kidney and brain mitochondria. Mitochondria isolated from kidney (A, B) or brain (C, D) were incubated as in Fig. 1A. Panels A and C show exemplar H_2_O_2_ traces ± FCCP or ± rotenone. (E) The rate of H_2_O_2_ production after addition of succinate to the closed chamber is shown for heart, kidney and brain mitochondria. (F) The rate of H_2_O_2_ production over the first 10 min following reoxygenation is shown for heart, kidney and brain mitochondria. * = p < 0.05, **** = p < 0.0001. Data are means ± SEM of *n* = 9–28 experiments each on a separate mitochondrial preparation.

Succinate accumulates during ischaemia in the heart, liver, kidney and brain [9], but its role in driving RET ROS upon reperfusion has mainly been explored in heart mitochondria [29]. Hence, we next assessed succinate-driven RET ROS in kidney (Fig. 3A and B) and brain (Fig. 3C and D) mitochondria. Prevention of this ROS production by rotenone and FCCP confirmed that it was due to RET (Fig. 3A–C). An increase in H_2_O_2_ production was observed immediately upon addition of succinate, followed by further H_2_O_2_ generation upon reoxygenation (Fig. 3B–D). The levels of initial H_2_O_2_ production (Fig. 3E) and that post-reoxygenation (Fig. 3F) were also determined and compared to those of heart mitochondria. The isolated mitochondria model can thus be extended to isolated mitochondria from other tissues and these data are consistent with succinate-driven RET ROS production contributing to IR injury in the kidney and the brain.

### 3.4 Assessing prevention of RET ROS by malonate

We next used our *in vitro* model to assess the effect of disodium malonate (DSM) at attenuating RET ROS upon reoxygenation. DSM has shown efficacy *in vivo* at preventing tissue damage in the heart and brain when administered intravenously (IV) upon reperfusion [[13], [14], [15]]. The efficacy of DSM in these *in vivo* models has been attributed to its rapid uptake into the cytosol via the monocarboxylate carrier 1 (MCT1) and from there via the dicarboxylate carrier into the mitochondrial matrix where it acts as a competitive inhibitor of SDH, preventing RET ROS upon reperfusion [13,15]. Here we tested this assumption *in vitro* under conditions mimicking reperfusion.

**Fig. 4:**
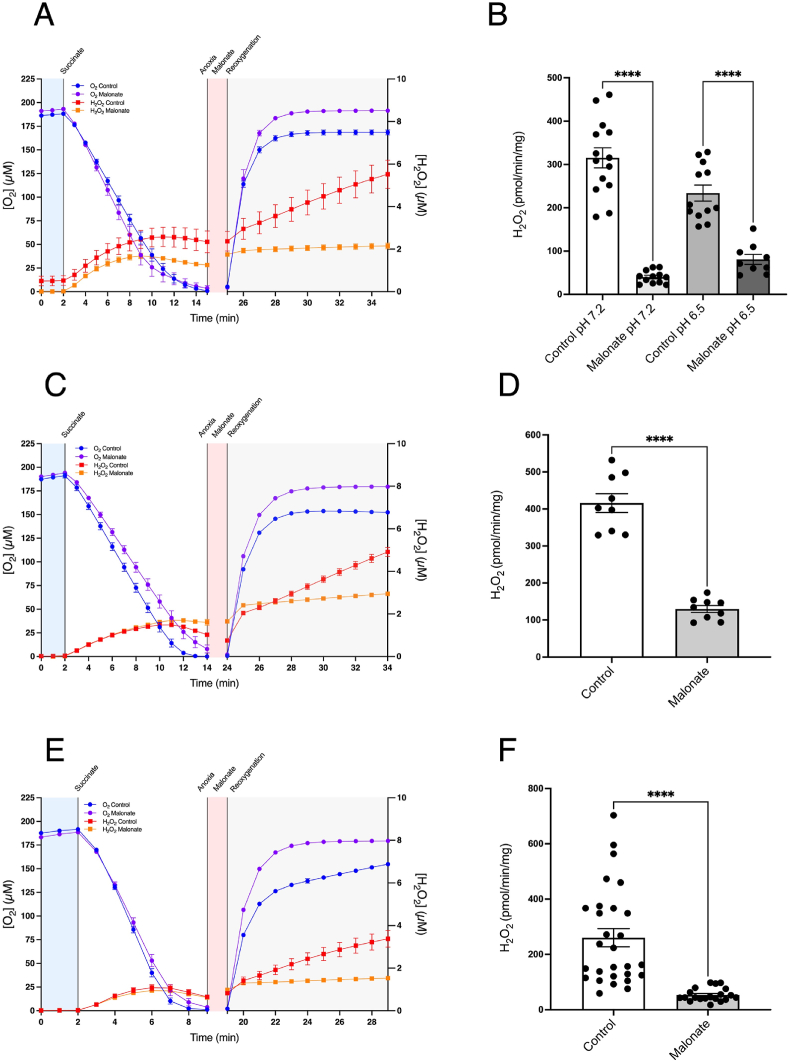
Inhibition of RET ROS upon reoxygenation by disodium malonate. Mitochondria isolated from heart (A, B), kidney (C, D) or brain (E, F) were incubated as in Fig. 1A. Where indicated disodium malonate (10 mM) was added after 5 min of anoxia. Panels A, C & E show time courses for each respective organ while panels B, D & F show the initial rate of H_2_O_2_ production over the first 10 min following reoxygenation. **** = p < 0.0001. Data are means ± SEM of *n* = 9–28 experiments each on a separate mitochondrial preparation.

To model the clinically relevant prophylactic administration of DSM immediately prior to reperfusion, we incubated heart mitochondria with succinate to generate anoxia (Fig. 4A), and after 5 min anoxia DSM was added and anoxia maintained for 5 min, followed by reoxygenation (Fig. 4A). Similar experiments were carried out at pH 6.5 and the initial slopes of H_2_O_2_ production upon reoxygenation ± DSM are shown in Fig. 4B. Similar experiments with kidney (Fig. 4C and D) and brain (Fig. 4E and F) mitochondria also showed that addition of malonate decreased H_2_O_2_ production upon reoxygenation and the initial slopes of H_2_O_2_ production following reoxygenation ± DSM are shown in Fig. 4D and F. These findings are consistent with IV DSM acting to prevent RET ROS in the heart, brain and kidney *in vivo* [13].

### *3.5* In vitro *screening of molecules to prevent RET ROS*

**Fig. 5:**
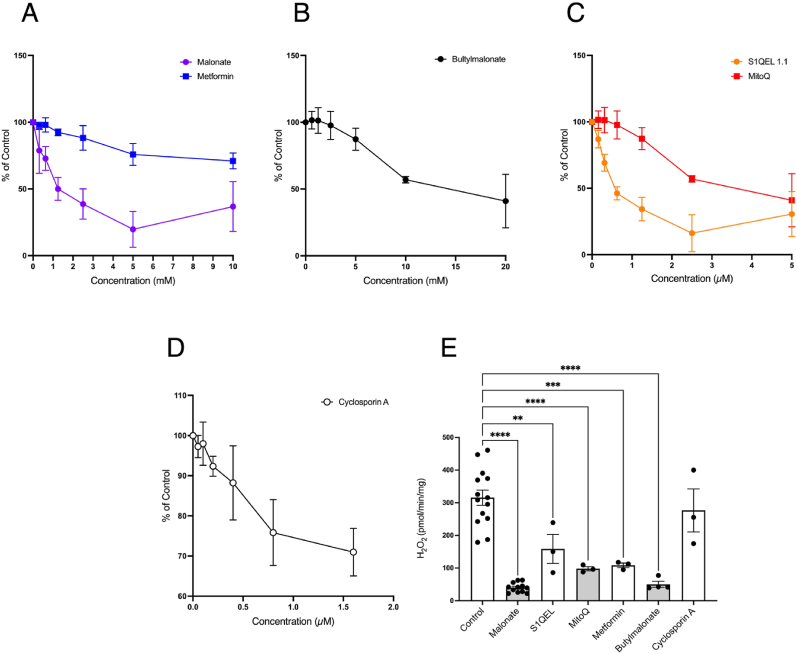
Effects of mitochondria-acting compounds on RET ROS *in vitro.* (A–D) Mitochondria isolated from the heart were incubated under the same conditions as described in Fig. 1A in a 96 well plate. Concentrations of malonate and metformin (0–10 mM, A), butylmalonate (0–20 mM, B), MitoQ and S1QEL 1.1 (0–5 μM, C) and Cyclosporin A (0–1.6, D) were assessed. The mean slopes ± SD for the rate of Amplex Red oxidation were determined in quadruplicate technical replicates at each concentration. (E) From the experiments shown in A-D the effects of malonate (10 mM), butylmalonate (20 mM), metformin (10 mM), S1QEL 1.1 (2.5 μM), MitoQ (2.5 μM) and Cyclosporin A (1.6 μM) were assessed on the rate of H_2_O_2_ production over the first 10 min following reoxygenation compared with control conditions. ** = p < 0.01, *** = p < 0.001, **** = p < 0.0001. Data for (E) are means ± SEM of *n* = 3–12 experiments each on a separate mitochondrial preparation.

We next assessed if our *in vitro* model could be used to screen for molecules to prevent RET ROS prior to *in vivo* testing. For this we chose a series of molecules known to impact mitochondrial function: butylmalonate, an inhibitor of the dicarboxylate carrier [40]; metformin, an inhibitor of complex I [41]; MitoQ, a mitochondria targeted antioxidant [42]; S1QEL 1.1, a compound that blocks RET ROS at complex I [18]; and Cyclosporin A that prevents induction of the mitochondrial permeability transition pore [43]. Before assessing the impact of these compounds on RET ROS upon reoxygenation, we first used a plate reader screen to generate dose-responses for their effects on RET ROS under ambient air conditions (Fig. 5A–D). In this assay, DSM was effective at a concentration relative to succinate known to be protective against IR injury in the heart *in vivo* [15]. From this we inferred compound doses for testing *in vitro* RET ROS upon reoxygenation following anoxia (Fig. 5E). Butylmalonate inhibited RET ROS by preventing uptake of succinate into mitochondria via the dicarboxylate carrier. Metformin also affected RET ROS, but this seems unlikely to be due to its direct impact on complex I from the matrix, as its uptake into mitochondria is unlikely to be fast. MitoQ affected RET ROS, most likely due to its interaction with the mitochondrial inner membrane slightly lowering Δp [29]. S1QEL 1.1 also affected RET ROS probably via its direct interaction with complex I [18], while Cyclosporin A did not impact ROS synthesis via RET. Hence this approach can be used to screen compounds for their impact on RET ROS prior to *in vivo* experiments.

### 3.6 Conclusion

While this paper was in review a publication appeared that also assessed ROS production in an isolated mitochondria model of IR injury [44]. Our results complement this publication and also extend our knowledge in a number of ways, such as by extension to mitochondria from other tissues (kidney and brain) and to enable an *in vitro* screen for drugs to attenuate ROS production in IR injury. One interesting difference between these two studies was that in that paper [44] a range of mitochondrial substrates were used whereas here we focussed on succinate. Our reason for investigating ROS driven by succinate was that *in vivo* in a range of tissues, including the heart [33,45], brain [14] and kidney [46], we have shown that the succinate accumulated during ischaemia is a major contributor to ROS upon reperfusion. However, the role of other mitochondrial metabolites in driving ROS production from other sites than complex I must also be considered [44]. Here we have established an *in vitro* system that enables us to assess mechanistic aspects of mitochondrial ROS production driven by succinate oxidation and RET during the IR injury that occurs at reperfusion. Using this model, we showed that there was a significant ROS burst upon reoxygenation of anoxic mitochondria at pH 6.5, which is likely to occur in ischemic tissues. Furthermore, we show that the low levels of ADP present in ischemic tissue are well below the threshold to decrease the Δp and thereby prevent RET ROS upon reperfusion. Attenuation of RET ROS via administration of DSM was observed using this system, in keeping with studies performed *in vivo*. Finally, we demonstrated that this approach could be used to screen for potential therapeutic compounds for IR injury prior to assessment in animal models.

## CRediT authorship contribution statement

**Annabel Sorby-Adams:** Writing – review & editing, Writing – original draft, Methodology, Investigation, Formal analysis, Data curation, Conceptualization. **Tracy A. Prime:** Writing – review & editing, Methodology, Investigation, Formal analysis, Data curation. **Jan Lj Miljkovic:** Writing – review & editing, Methodology, Investigation, Data curation. **Hiran A. Prag:** Writing – review & editing, Writing – original draft, Supervision, Formal analysis, Conceptualization. **Thomas Krieg:** Writing – review & editing, Supervision, Project administration, Conceptualization. **Michael P. Murphy:** Writing – review & editing, Writing – original draft, Supervision, Project administration, Funding acquisition, Formal analysis, Conceptualization.

## Declaration of competing interest

The authors declare the following financial interests/personal relationships which may be considered as potential competing interests: Michael P. Murphy is on the Scientific Advisory Board of MitoQ, Inc. and holds stock in the company, is CSO of Camoxis Therapeutics Inc., which is developing malonate as a therapy and he holds patents in therapeutic applications of malonate. Thomas Krieg is CMO of Camoxis Therapeutics Inc., which is developing malonate as a therapy and he holds patents in therapeutic applications of malonate. Hiran A. Prag holds a patent in therapeutic applications of malonate. All other authors declare no conflicts of interest.

## Data Availability

Data will be made available on request.
