# Corrigendum to “A model of mitochondrial superoxide production during ischaemia-reperfusion injury for therapeutic development and mechanistic understanding” [Redox Biol. 72 (2024) 103161]

**DOI:** 10.1016/j.redox.2024.103210

**Published:** 2024-05-28

**Authors:** Annabel Sorby-Adams, Tracy A. Prime, Jan Lj Miljkovic, Hiran A. Prag, Thomas Krieg, Michael P. Murphy

**Affiliations:** a MRC Mitochondrial Biology Unit, University of Cambridge, The Keith Peters Building, Cambridge, CB2 0XY, UK; b Department of Medicine, University of Cambridge, Hills Road, Cambridge, CB2 0QQ, UK

The authors regret that Fig. 1E was incorrect. The correct Fig. 1E is below.

**Figure:**
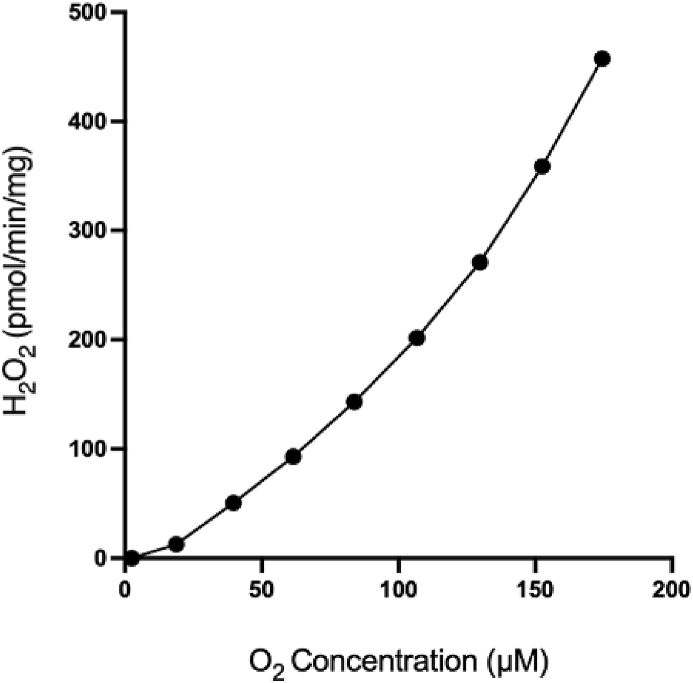

The authors would like to apologise for any inconvenience caused.

