# Supplementary material for: A model of mitochondrial superoxide production during ischaemia-reperfusion injury for therapeutic development and mechanistic understanding
Source: Redox Biol. 2024 Apr 24;72:103161. doi: 10.1016/j.redox.2024.103161 (PMC11066467; doi:10.1016/j.redox.2024.103161)
Supplement: Multimedia component 1 [file mmc1.docx]

**A model of mitochondrial superoxide production during ischaemia-reperfusion injury for therapeutic development and mechanistic understanding**

Annabel Sorby-Adams^a^, Tracy A. Prime^a^, Jan Lj. Miljkovic^a^, Hiran A. Prag^b^, Thomas Krieg^b^ and Michael P. Murphy^a,b,*^

^a^ *MRC Mitochondrial Biology Unit, University of Cambridge, The Keith Peters Building, Cambridge CB2 0XY, UK*

^b^ *Department of Medicine, University of Cambridge, Hills Road, Cambridge CB2 0QQ, UK*

**
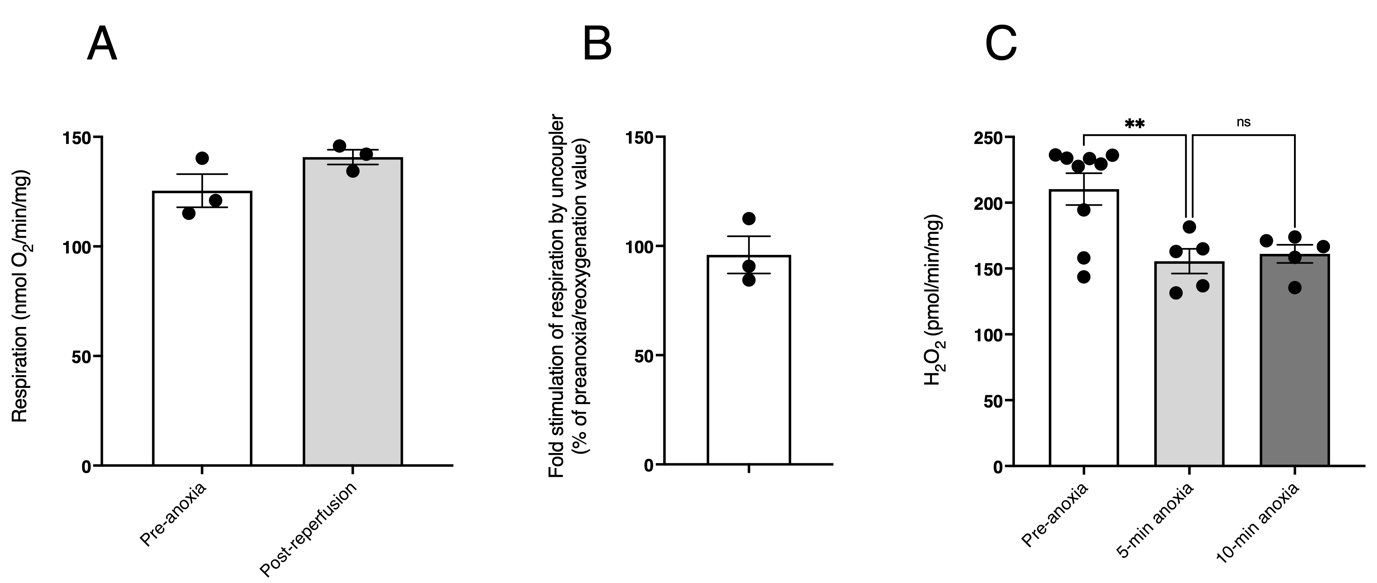
**

**Supplemental Fig. 1. Effects of incubation duration on mitochondrial coupling and RET ROS *in vitro.*** Mitochondria isolated from the heart were incubated and analysed as described in Fig. 1A. (A) Mitochondrial coupled respiration rate was measured in a closed Oroboros incubation chamber prior to anoxia. Then, after 10 min anoxia the chamber was reoxygenated and sealed so that respiration rate could again be measured. (B) Mitochondrial coupled respiration rate was measured in a closed Oroboros incubation chamber prior to anoxia and then uncoupled respiration rate was measured by addition of FCCP (500 nM) and the fold-stimulation of respiration calculated. In separate experiments, mitochondria were incubated and after 10 min anoxia the chamber was reoxygenated and sealed so that coupled respiration rate could be measured, followed by addition of FCCP and the fold-stimulation of respiration calculated and is shown as a percentage of that prior to anoxia. (C) The initial rates of H_2_O_2_ production after addition of succinate was determined in a closed Oroboros incubation chamber prior to anoxia. Then, after 5- or 10-min anoxia the chamber was reoxygenated and the rates of H_2_O_2_ production measured. Data are means ± SEM of *n* = 3-10 experiments each on a separate mitochondrial preparation. **p<0.01.
